# Effects of pretreatment with a combination of ultrasound and γ-aminobutyric acid on polyphenol metabolites and metabolic pathways in mung bean sprouts

**DOI:** 10.3389/fnut.2022.1081351

**Published:** 2023-01-10

**Authors:** Lidong Wang, Xiaoqiang Li, Fei Gao, Ying Liu, Shuangjing Lang, Changyuan Wang

**Affiliations:** ^1^College of Food Science, Heilongjiang Bayi Agricultural University, Daqing, China; ^2^Daqing Center of Inspection and Testing for Agricultural Products and Processed Products Ministry of Agriculture and Rural Affairs, Heilongjiang Bayi Agricultural University, Daqing, China; ^3^Department of National Coarse Cereals Engineering Research Center, Heilongjiang Bayi Agricultural University, Daqing, China

**Keywords:** polyphenols, antioxidant capacity, untargeted metabolomics, differential metabolites, metabolic pathways

## Abstract

**Background:**

Polyphenols play an important role in human nutrition, therefore, how to improve its content with innovative approach is important, and understanding the metabolic pathys is necessary. Mung beans are rich in polyphenols, which made them have physiological functions such as hypoglycemia, antioxidant, and hypotension. However, the content of polyphenols in natural mung bean is relatively low, and it needs to be increased. The methods of increasing polyphenol content in grains and beans by enrichment include physical stress, such as ultrasonic stress, hypoxia stress and ultraviolet radiation, and single exogenous substance stress, such as exogenous amino acids, exogenous sugars. But, the enrichment of polyphenols using exogenous substances in combination with physical stress is less applied. Therefore, this study innovated the use of exogenous γ-aminobutyric acid (GABA) combined with ultrasonic stress to enrich mung bean sprouts polyphenols and enhance their content. The metabolic pathways of the enrichment process were also analyzed to provide a reference for studies related to the enrichment of polyphenols.

**Methods:**

Mung bean seeds were pretreated with a combination of ultrasound and GABA under different conditions. Single-factor test and response surface methodology were used for optimizing pretreatment conditions of mung bean. Effects of combined pretreatments on the polyphenols content and antioxidant activity of sprouted mung beans were investigated. Additionally, metabolites were identified, and metabolic pathways were analyzed using non-targeted metabolomics techniques.

**Results:**

Optimal conditions of mung bean pretreatment were found to be 370 W for ultrasound power, 40 min for ultrasonication time, 10 mmol/L for GABA concentration, and 8 h for the soaking duration. Under these conditions, the predicted polyphenol content was found to be 4.52 mg GAE/g DW. The pretreatment of mung beans with a combination of ultrasound and exogenous GABA resulted in mung bean sprouts with enhanced polyphenol content and antioxidant activity compared to mung beans germinated without pretreatment. A significant increase in the content of six polyphenols [Genistein, (-)-Epigallocatechin, Epicatechin, Nobiletin, Naringenin, Biochanin A] in the pretreated and germinated mung beans was found, and six metabolic pathways (flavonoid biosynthesis, isoflavones biosynthesis, biosynthesis of phenylpropanoids, anthocyanin biosynthesis, biosynthesis of secondary metabolites, and metabolic pathways) were significantly activated.

**Conclusion:**

The obtained results suggest that a combination of ultrasound and exogenous GABA treatment can be used to produce mung bean sprouts with enriched polyphenols content and enhanced antioxidant activity.

## 1. Introduction

Mung beans (*Vigna radiata*) are small and green seeds belong to the legume family with a wide range of food applications (1). According to Chinese medicine, mung beans are thought to help in the body’s detoxification, reduction of skin irritation, and clearing of body heat (2). Mung beans are rich in essential nutrients such as protein and starch (3). Also, mung beans contain bioactive substances such as phenolic acids and flavonoids with potential health benefits (4).

People are being pushed toward a healthy diet by the rise in health problems. Mung beans and their sprouts have received increasing attention for their high nutritional value. Sprouting can significantly reduce antinutrients content of mung beans while the content of beneficial substances such as polyphenols and dietary fiber can be enhanced (5). Treatments with magnetic field (6), ultrasound (7), UV irradiation (8), vacuum stress (9), and exogenous amino acids (10) were found to enhance the content of bioactive compounds such as polyphenols of grain and seed sprouts. For example, the cavitation and thixotropic effects of low-intensity ultrasound can enhance the penetration of cell wall and cell membrane, enzymes activity, the rate of material exchange and metabolism inside and outside the cell, and the growth and development of plants (11). Besides, there is a relationship between γ-aminobutyric acid (GABA) and the antioxidant system of plants. The GABA has an important role in growth and stress tolerance, signaling activation, regulation of protein degradation, and hormone synthesis in plants, as well as phenolic enrichment (12). Many studies have reported. Effect of treatment with exogenous sucrose (13), plasma-activated water (14), UV-B treatment (15), NaCl and glucose (16), sodium citrate, sodium acetate and sodium tartrate (17), Ca^2+^ (18), polyamines (19), spermidineon (20) germination quality, and active substances of mung bean sprouts. Although effects of germination on the phenolics content of legumes have been extensively studied, research performed to investigate effect of a combination of pretreatments on the polyphenols content of legumes during germination and to understand underlying mechanisms are limited. To the best of our knowledge, no scholars have studied the combination of ultrasound and exogenous GABA treatment of mung beans or other grain beans on the content of active substances and metabolic pathways. This study aimed to investigate effects of treatment with a combination of ultrasound and GABA pretreatment on the polyphenols content of mung beans sprouts. Differential polyphenol metabolites and metabolic pathways were also analyzed and identified. Treatment conditions were optimized to produce mung bean sprouts with enriched polyphenols content and higher antioxidant activity.

## 2. Materials and methods

### 2.1. Materials

Total Antioxidant Kit, ABTS, 2,2-diphenyl-1-picrylhydrazyl (DPPH), potassium persulfate, ethanol, formic acid, ammonium formate, and acetonitrile (Sigma-Aldrich, Shanghai, China). Folin-ciocalteu reagent, rutin, sodium carbonate, aluminum chloride, sodium nitrite, γ-aminobutyric acid, gallic acid, and sodium hypochlorite (Shanghai Macklin Biochemical Technology Co., Ltd., Shanghai, China). Other chemicals and reagents were of analytical grade.

### 2.2. Germination of mung beans

Mung bean seeds were obtained from Beidahuang Grain Group Co., Ltd. Seeds (50.00 g) with granular shapes and uniform sizes were chosen for the study. Surface of the mung bean seeds was disinfected by immersion in 0.5% (v/v) sodium hypochlorite solution for 15 min, and then rinsed three times with distilled water and drained. The treated mung bean seeds were placed in a 250 mL beaker, and GABA solution was added at a seed/liquid ratio of 1:3 (w/v). The samples were placed in an ultrasonic generator (KH-500GDV, Hechuang Ultrasonic Instrument Co., Ltd., Kunshan, China) at 20°C with a specific power for 40 min, and then transferred to a 30°C water bath. The soaked mung beans were placed in the germination tray and 500 mL of GABA solution was placed at the bottom of the germination tray, and the seeds were covered with two layers of gauze. The samples were placed in an incubator (ZXMP-R1230, Zhicheng Inc., Shanghai, China) at 30°C and a humidity of 75% for 48 h, and the GABA medium was replaced every 12 h. The mung bean sprouts were washed with distilled water to remove mucus, freeze-dried to constant weight, crushed three times, packaged in vacuum bags, and stored in a freezer at −80°C until use.

### 2.3. Single-factor experiment

A single-factor experiment was carried out to investigate the effects of ultrasound and GABA parameters on extraction efficiency and to determine a narrow range of different parameters. Four extraction parameters were studied, including GABA concentration (0, 5, 10, 15, and 20 mmol/L), GABA soaking time (2, 4, 6, 8 and 10 h), ultrasonic power (240, 280, 320, 360, and 400 W) and ultrasonic time (10, 20, 30, 40, and 50 min).

### 2.4. Response surface optimization experiment

According to results of the single-factor experiment, three main variables (GABA concentration, ultrasonic power, and ultrasonic time) were chosen for the response surface optimization. Design Expert Software (version 8.0.6.1, Stat-Ease, Inc., Minneapolis, USA) was used to generate a Box-Behnken design and analyze the data. A 3-level, 3-factor Box-Behnken design was generated by the software, consisting of 17 experimental runs (21). Each variable was coded at three levels (−1, 0, 1). The experimental factors and levels are listed in Table 1.

**TABLE 1:** Coded levels of independent variables.

Independent variables	Coded units	Coded levels		
		−1	0	1
GABA concentration (mmol/L)	X_1_	5	10	15
Ultrasonic power (W)	X_2_	320	360	400
Ultrasonic time (min)	X_3_	10	30	50

### 2.5. SEM analysis

Ultrasonic treatment can effectively activate various enzymatic activities and accelerate the germination process. It promotes the decomposition of large molecules such as starch and protein into small molecule compounds. Scanning electron microscopy (SEM) was used to observe the changes of the apparent morphology after pretreatment germination. SEM analysis carried out as described by Wang (22). Powder particles morphology was analyzed using a SEM (S-3400N, Hitachi Limited, Tokyo, Japan) at magnification of ×5000.

### 2.6. Extraction of mung bean sprouts

Optimal conditions for extraction of sprouts using ethanol were chosen based on a preliminary study. A sample of 20.00 g mung bean sprout powder was weighed into a 1,000 mL conical flask with a stopper and connected with a water-cooling device. Aqueous ethanol solution (60%, v/v) was added at a solid-to-liquid ratio of 1:35 (w/v) and mixed well. The mixture was stirred at a temperature of 40°C for 2 h using a magnetic stirrer. The extract was obtained by vacuum filtration. Each sample was extracted three times in parallel and stored in a refrigerator at 4°C for determination of free polyphenols, free flavonoids content, antioxidant activity, and untargeted metabolomics assays.

### 2.7. Determination of free polyphenols and free flavonoids

The free polyphenols content was determined by the Folin-ciocalteu method (23). A sample of 1.0 mL extract was pipetted into a test tube, 3.0 mL of Folin-ciocalteu reagent was added, shaken well, and allowed to stand for 30 s. Then, 6.0 mL of 12% Na_2_CO_3_ solution was added, the mixture shaken well, and diluted to 25 mL. The mixture was stored in the dark at 25°C for 2 h, and the absorbance was measured at a wavelength of 765 nm using a spectrophotometer (TU-1800 UV, Beijing general analytical instrument, Beijing, China). The standard curve equation of gallic acid was as follows: y = 0.15x + 0.059 (*R*^2^ = 0.9997). The results were recorded as mg of gallic acid equivalents per g dry weight (mg GAE/g DW) of mung bean sprouts.

The content of free flavonoids was determined with rutin as a standard (24). A sample of 3.0 mL extract was put into a 10 mL graduated test tube, and 0.3 mL of 5% NaNO_2_ was added. The mixture was shaken well and allowed to react in the dark for 6 min, and 0.3 mL of 10% Al (NO_3_)_3_ was added, mixed well, and allowed to react in the dark again for 6 min. Finally, 4 mL of 4% NaOH solution was added, and the mixture was diluted to 10 mL using 70% ethanol solution, mixed well, and allowed to react in the dark for 15 min. The absorbance was then measured at a wavelength of 510 nm. The standard curve equation of rutin was as follows: y = 11.817x + 0.0362 (*R*^2^ = 0.9992) of mung bean Sprouts. The data were expressed as mg rutin equivalents per g dry weight (mg RE/g DW) of mung bean sprouts.

### 2.8. Antioxidant activity assays

#### 2.8.1. Total antioxidant capacity

The total antioxidant capacity of mung bean sprouts extract was determined using a T-AOC kit. The absorbance was measured at 520 nm (TU-1800 UV, Beijing general analyical instrument, Beijing, China). The total antioxidant capacity was calculated as follows:


(1) $$T-AOC=0.01\times \frac{\text{O}D_{1}-OD_{2}}{30}\times \frac{V_{1}}{V_{2}}$$

Where OD_1_ is the absorbance value of the sample to be tested. OD_2_ is the absorbance value of the control sample. V_1_ is the volume of the reaction solution. V_2_ is the sampling amount.

#### 2.8.2. DPPH radical scavenging capacity

A sample of 0.40 mL extract was mixed with 2.6 mL of DPPH in ethanol solution (0.1 mmol/L, w/v) and placed in the dark for 30 min. Then, the absorbance was measured at 517 nm using absolute ethanol as a blank (25). The DPPH radical scavenging capacity was calculated as follows:


(2) $$RSC=\left(1-\frac{A_{1}-A_{2}}{A_{0}}\right)\times 100\%$$

Where A_1_ is the absorbance of 400 μL sample extract and 2.6 mL DPPH solution. A_2_ is the absorbance of 400 μL extract and 2.6 mL absolute ethanol. A_0_ is the absorbance of 400 μL absolute ethanol and 2.6 mL DPPH solution Control absorbance value of the solution.

#### 2.8.3. ABTS radical scavenging capacity

A sample of 0.1 g ABTS and 0.029 g potassium persulfate powder was dissolved in deionized water to make 100 mL of ABTS free radical stock solution. The prepared solution was stored in a refrigerator at 4°C for 12 h and then was diluted until the absorbance at 734 nm was 0.700 ± 0.020. A sample of 0.2 mL extract was put in a test tube and 5.8 mL of ABTS solution was added, mixed well, and the mixture was allowed to react in the dark for 6 min. Then, the absorbance was measured at 734 nm (26). The ABTS radical scavenging activity was calculated as follows:


(3) $$RSC=\left(\frac{A_{1}-A_{2}}{A_{1}}\right)\times 100\%$$

Where A_1_ is the absorbance value of the blank control group. A_2_ is the absorbance value of the sample solution measurement group.

### 2.9. Untargeted metabolomic analysis

Untargeted metabolomics was performed with reference to Gabriele’s method (27). The extract was centrifuged at 12,000 rpm for 10 min at 4°C, and the supernatant was filtered through a 0.22 μm membrane. The LC analysis was performed on a Vanquish UHPLC System (Thermo Fisher Scientific, Waltham, USA). Chromatography was carried out with an ACQUITY UPLC^®^ HSS T3 column (150 × 2.1 mm, 1.8 μm) (Waters, Milford, MA, USA). Chromatographic conditions were chromatographic column, a flow rate of 0.25 mL/min, column temperature of 40°C, and injection volume of 2 μL. In the positive ion mode, the mobile phases were 0.1% formic acid acetonitrile (C) and 0.1% formic acid water (D), and in negative ion mode, the mobile phases were acetonitrile (A) and 5 mM ammonium formate water (B) (28). Mass spectrometry conditions were as follows: Thermo Orbitrap Exploris 120 mass detector (Thermo Fisher Scientific, Waltham, USA), electrospray ionization source (ESI), and data were collected in positive and negative ion modes. The first-level full scan was performed with a resolution of 60,000, the first-level ion scanning range was m/z 100–1000, and the second-level fragmentation was performed by HCD. The collision voltage was 30%, and the second-level resolution was 15,000. The first four ions were fragmented. At the same time, dynamic exclusion was used to remove unnecessary MS/MS information (29).

### 2.10. Statistical analysis

All experiments were carried out at least in triplicate, and the data were statistically analyzed and expressed as mean ± SD. Analysis of variance (ANOVA) and Duncan’s multiple range test with a confidence interval of 95% was performed using SPSS 26 software (SPSS 26, International Business Machines Corporation, Armonk, New York, USA).

Data processing and multivariate analysis: The raw data were firstly converted to mzXML format by MSConvert in ProteoWizard software package (v3.0.8789) and processed using XCMS for feature detection, retention time correction, and alignment. The metabolites were identified by accuracy mass (<30 ppm) and MS/MS data which were matched with HMDB^1^, massbank^2^, LipidMaps^3^, mzcloud^4^, and KEGG^5^. Data were mean-centered using scaling. Models were built on principal component analysis (PCA), orthogonal partial least-square discriminant analysis (PLS-DA), and partial least-square discriminant analysis (OPLS-DA). *P* value < 0.05 and VIP values > 1 were considered to be statistically significant metabolites.

Pathway analysis: Differential metabolites were subjected to pathway analysis by MetaboAnalyst. The metabolites and corresponding pathways were visualized using KEGG Mapper tool.

## 3. Results

### 3.1. Single-factor analysis

**FIGURE 1:**
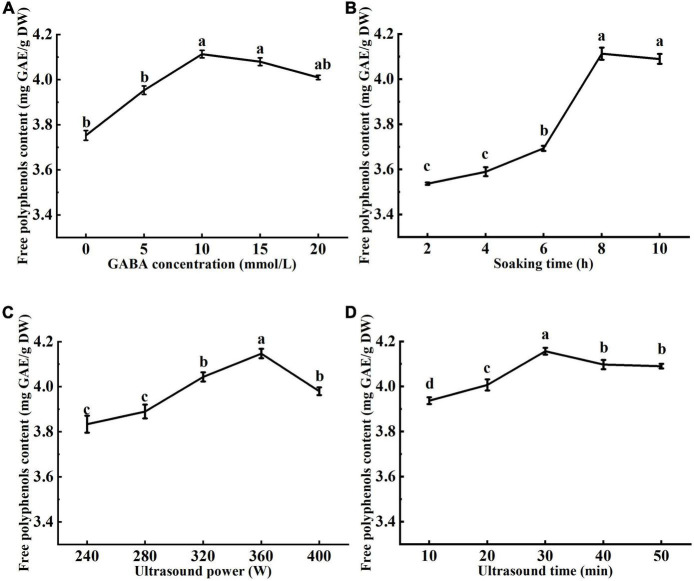
Effects of γ-aminobutyric acid (GABA) concentration **(A)**, soaking time **(B)**, ultrasonic power **(C)**, and ultrasonic time **(D)** on the free polyphenol content of mung bean. The GABA concentration, soaking time, ultrasonic power, and ultrasonic time were 10 mmol/L, 8 h, 360 W, and 30 min, respectively. Data are mean ± SD (*n* = 3). Different subscript letters indicate significant differences at *p* < 0.05.

Effects of ultrasound and GABA conditions, including GABA concentration, GABA soaking time, ultrasound power, and ultrasound treatment time on the free polyphenols content are shown in Figure 1. The free polyphenols content increased with the increase of GABA concentration up to 10 mmol/L (Figure 1A). At GABA concentration of 10 mmol/L, the highest free polyphenol content was 4.11 mg GAE/g DW. However, as the GABA concentration increased to 15 and 20 mmol/L, the free polyphenols content tended to decrease. It was reported that GABA as a signaling molecule binds to glutamate receptors in plant cells (30). In this study, with the increase of GABA concentration, GABA bonded to the receptor to saturation, increasing the free polyphenols content. From Figure 1B, it can be seen the highest free polyphenol content was achieved when mung beans were soaked in GABA solution for 8 h. This may be attributed to that ultrasonic pretreatment caused changes in the structure of cell membranes, increasing GABA solution absorption capacity of the mung bean seeds, which reflected in increased free polyphenols content (31). When mung beans are soaked at appropriate temperature for enough time, the cell wall is soften, enhancing seed coat permeability, and releasing seed dormancy (32). GABA in solution penetrated the seeds and involved in the anabolism of polyphenols by activating related enzymes. However, the free polyphenol content decreased as soking prolonged to 10 h, which may be attributed to the loss of polyphenols and reduction of enzymes activity as soaking prolonged. A shorter soaking time is not enough to soften the seed coat and the embryonic axis is difficult to grow through the seed coat. Therefore, the soaking time of 8 h was chosen as optimal.

The cavitation of ultrasound produces tiny bubbles, which collapse at the mung bean seed coat, damaging the surface of the seed coat, and creating tiny holes. The seed coat is a physical barrier that prevents the seed from absorbing water and oxygen, which are necessary for seed germination. Therefore, increasing seed coat porosity may increase water and oxygen uptake and promote germination (33). It can be seen from Figure 1C that the free phenol content firstly increased then decreased with the increase of ultrasonic power. This may be explained by that the low ultrasonic power promoted hydration of the mung bean seeds and enhanced activity of polyphenol metabolic pathway enzymes (34, 35), accelerating synthesis of polyphenols and the release of bound phenolic compounds. Ultrasonication at 400 W resulted in a decrease in the polyphenol content. It may be attributed to mechanical damage and cavitation of high ultrasonic power, which resulted in structural breakage of mung bean seeds and loss of polyphenols. From Figure 1D, with the increase of sonication time, the free polyphenol content firstly increased reaching a maximum value at 30 min of ultrasonication, then tended to decrease. This may be explained by that ultrasonication for shorter time can effectively activate enzymes of polyphenol metabolic pathway and promotes the release of bound phenolics from the seed coat. However, the prolonged sonication produces damage to seed cells, reduces polyphenol synthesis, and enhance the loss of polyphenols in the soaking solution.

### 3.2. Response surface optimization

#### 3.2.1. Effects of ultrasonic and GABA on the free polyphenols content

The response surface optimization design factor levels and results are shown in Tables 1, 2. Response surface results were analyzed with Design Expert 8 software, and the ANOVA results are shown in Table 3. The total model was highly significant (*p* < 0.01), the lack of fit was not significant (*p* > 0.05). The results showed that the polynomial has acceptable accuracy, it was possible to predict effects of GABA concentration, ultrasonic power, and ultrasonic time on the free polyphenol content of mung beans after germination. A significance test of the regression model showed that X_1_, X_1_^2^, and X_2_^2^ had a significant effect on polyphenols content (*p* < 0.05), and X_2_, X_3_, X_1_X_2_, and X_3_^2^ had a highly significant effect on the polyphenols content (*p* < 0.01). However, effect of other factors was not significant. According the *F* value, effect of factors on the polyphenol content of mung bean sprouts was in the order ultrasound power > ultrasound time > GABA concentration.

**TABLE 2:** Response surface design and free polyphenols content of the mung bean extracts.

Run	X_1_ (mmol/L)	X_2_ (W)	X_3_ (min)	FP content (mg GAE/g DW)
1	0 (10)	0 (360)	0 (30)	4.59 ± 0.04
2	0 (10)	0 (360)	0 (30)	4.44 ± 0.06
3	−1 (5)	0 (360)	1 (50)	4.25 ± 0.03
4	−1 (5)	0 (360)	−1 (10)	4.15 ± 0.02
5	0 (10)	1 (400)	−1 (10)	4.23 ± 0.04
6	1 (15)	0 (360)	−1 (10)	4.19 ± 0.01
7	0 (10)	0 (360)	0 (30)	4.43 ± 0.06
8	0 (10)	0 (360)	0 (30)	4.54 ± 0.03
9	1 (15)	−1 (320)	0 (30)	4.22 ± 0.05
10	0 (10)	−1 (320)	−1 (10)	3.97 ± 0.02
11	0 (10)	1 (400)	1 (50)	4.36 ± 0.01
12	0 (10)	0 (360)	0 (30)	4.39 ± 0.06
13	1 (15)	0 (360)	1 (50)	4.48 ± 0.03
14	−1 (5)	−1 (320)	0 (30)	3.84 ± 0.01
15	−1 (5)	1 (400)	0 (30)	4.31 ± 0.05
16	1 (15)	1 (400)	0 (30)	4.13 ± 0.04
17	0 (10)	−1 (320)	1 (50)	4.13 ± 0.05

Box-Behnken design was used to optimize the conditions for enrichment of mung bean with polyphenols (Table 2), and a regression model was established as follows: three independent variables, including GABA concentration (X_1_), ultrasonic power (X_2_), and ultrasonic time (X_3_), on the free polyphenol contents, are represented.

**TABLE 3:** Analysis of variance (ANOVA) of response surface regression model.

Source	Sum of square	df	Mean square	*F*-value	Prob > *F*	Significance
Model	0.61	9	0.067	15.08	0.0009	**
X_1_	0.028	1	0.028	6.18	0.0418	*
X_2_	0.095	1	0.095	21.19	0.0025	**
X_3_	0.058	1	0.058	12.95	0.0088	**
X_1_X_2_	0.08	1	0.078	17.56	0.0041	**
X_1_X_3_	0.009025	1	0.009025	2.02	0.1981	
X_2_X_3_	0.000225	1	0.0002250	0.050	0.8288	
X_1_^2^	0.070	1	0.070	15.69	0.0055	**
X_2_^2^	0.21	1	0.21	47.32	0.0002	**
X_3_^2^	0.028	1	0.028	6.26	0.0408	*
Residual	0.031	7	0.004465			
Lack of fit	0.00375	3	0.001125	0.16	0.9170	
Pure error	0.028	4	0.006970			
Corrected total	0.64	16				

*Significant (*p* < 0.05), **extremely significant (*p* < 0.01).


$$\begin{array}{l} Y=4.48+0.059X_{1}+0.11X_{2}+0.085X_{3}-0.14X_{1}X_{2}\\ \;-0.048X_{1}X_{3}-0.0075X_{2}X_{3}-0.13X_{1}{}^{2}-0.22X_{2}{}^{2}\\ \;-0.081X_{3}{}^{2} \end{array}$$

#### 3.2.2. Response surface interaction analysis

**FIGURE 2:**
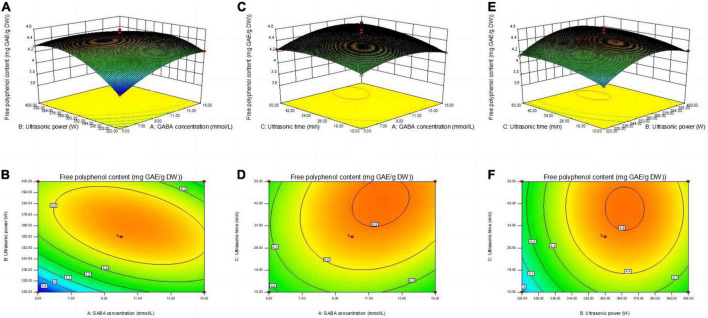
Contour lines and response surface diagrams of the interaction between X_1_X_2_ **(A,B)**, X_1_X_3_ **(C,D)**, and X_2_X_3_ **(E,F)**. (X_1_) GABA concentration, (X_2_) ultrasonic power, and (X_3_) ultrasonic time.

Analysis of the response surface map is based on a rational experimental design. Using the variability of the response surface and the sparsity of the contours, optimal process conditions can be determined while allowing visual evaluation of the interactions between factors. Changes in the response surface and contours in Figure 2 reflect the effect of interaction between GABA concentration (A) ultrasound power (B) and ultrasound time (C) on the free polyphenols content of germinated mung beans. The shape of the contour map reflects the interaction between various factors (13). When the contour is circular, this means that the interaction of the two factors is not significant, while elliptical or saddle-shaped contour indicates significant interaction between the two factors.

Figure 2 shows the response surface and contour plots for the interaction of AB (GABA concentration and ultrasound power), AC (GABA concentration and ultrasound time), and BC (ultrasound power and ultrasound time), where the AB interaction was significant (*p* < 0.05, Table 2). The contour plots of AB are elliptical (Figures 2A, B), indicating a strong interaction between the relevant factors, while the contour plots of AC and BC are circular, indicating a relatively weak interaction between factors.

#### 3.2.2. Determination and verification of optimal conditions

Process parameters were optimized using Design Expert 8 software. The optimal conditions for enrichment of mung bean sprout with polyphenols was obtained as follows: ultrasound power of 366.13 W, ultrasound time of 41.77 min, and GABA concentration of 11.27 mmol/L. The predicted polyphenols content was 4.52 mg GAE/g DW. As these parameters were not conducive to operation, parameters were adjusted to be ultrasound power 370 W, ultrasound time 40 min, and GABA concentration of 10 mmol/L. Validation tests were performed under these conditions (three times in parallel). Polyphenols content of 4.49 mg GAE/g was obtained for the mung bean sprouts, which was similar to the predicted value. This indicates that the optimized conditions for enrichment of mung bean sprout with polyphenols using response surface analysis are reliable.

### 3.3. Morphological structure of mung bean sprout powder

**FIGURE 3:**
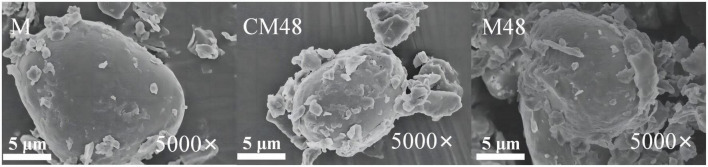
Scanning electron microscopy (SEM) images of the ungerminated and germinated mung beans. M, ungerminated mung bean; CM48, mung bean germinated for 48 h; M48, mung bean pretreated with a combination of ultrasonic and γ-aminobutyric acid (GABA) and germinated for 48 h.

Surface morphology of mung bean sprouts powder is shown in Figure 3. It can be seen that surface of the untreated mung bean powder is smooth with a small number of attached fine particles. However, surface of the germinated mung bean powder is disrupted with scaly and lamellar structure. This may be attributed to damage and breakdown in surface of starch granules and protein during germination (36). Besides, the cavitation and mechanical effects of ultrasound treatment might contribute to changes in morphological structure of mung bean sprouts particles (37).

### 3.4. Free polyphenols, flavonoids content, and antioxidant activity

**FIGURE 4:**
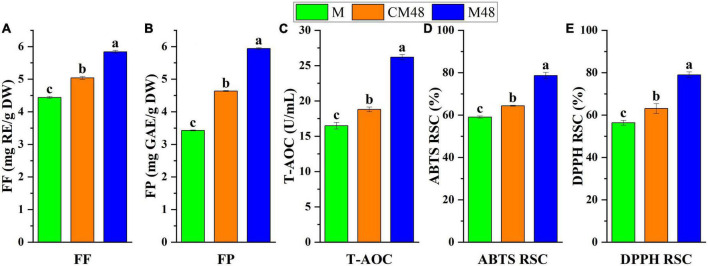
The free flavonoids content **(A)**, free polyphenols content **(B)**, and total antioxidant capacity **(C)**, ABTS radical scavenging activity **(D)**, and DPPH radical scavenging activity **(E)** of sprouted mung bean. M, mung bean; CM48, mung bean germinated for 48 h; M48, mung bean pretreated with a combination of ultrasonic and γ-aminobutyric acid (GABA) germinated for 48 h; FF, free flavonoids; FP, free polyphenols; T-AOC, total antioxidant capacity; ABTS, 2,2-Azino-bis-3-etilbenzotiazolin-6-sulfonic acid; DPPH, 2,2-diphenyl-1-picrylhydrazyl; RSC, radical scavenging capacity.

The free polyphenols and flavonoids content, total antioxidant capacity, ABTS free radical scavenging, and DPPH free radical scavenging activities of the ungerminated and germinated mung bean seeds are shown in Figures 4A–E. A combination treatment with ultrasound and GABA resulted in mung bean sprouts with enhanced free polyphenols and flavonoids contents compared to both the untreated mung bean seeds and seeds germinated without ultrasonic and GABA treatments.

Ultrasonic makes the seed shell fragment and accelerates the hydration process, leading to changes in the molecular structure and catalysis of enzymes, triggering the defense response system and enhancing the production of secondary metabolites such as polyphenols (38). Also, the cavitation and mechanical effects of ultrasonics enhance the permeability of cell membranes, promoting the diffusion and membrane transport of ions and metabolites (39). The exogenous GABA can pass through the cell membrane and enter the cell. Effect of GABA can be attributed to that it regulates the key enzyme genes of polyphenols synthesis, increasing protein expression and enzyme activity (40), stimulate hormone production, regulate growth and development, and further cause intracellular physiological and biochemical changes, regulating polyphenols metabolism pathway gene expression to achieve the enrichment effect. Therefore, the increase in polyphenols content of mung beans can be attributed to the combined effect of GABA, ultrasonic, and germination treatments.

The highest total antioxidant capacity, ABTS radical scavenging activity, and DPPH radical scavenging activity were found for mung bean subjected to a combination of GABA and ultrasound treatments and germinated for 48 h (Figures 3C–E). Polyphenolic compounds are natural antioxidants, therefore, the correlation between the free polyphenols and flavonoids content and antioxidant activities of the mung bean sprout extracts was analyzed. From Table 4, it can be seen that all correlation coefficients were above 0.946. The correlation between flavonoids and antioxidant capacity was higher. This is because the synthesis of flavonoids is downstream of the phenolic acid synthesis pathway and accumulates in larger quantities. These results indicate that the antioxidant activity is generally consistent with the polyphenols content, and the enhancement in antioxidant activity can be mainly attributed to the increase in antioxidants content such as polyphenols after GABA, ultrasound, and germination treatments of mung bean. Additionally, the antioxidant capabilities of mung beans may be influenced by the presence of vitamins such as vitamins E and C as well as other antioxidants (41). The contents of these substances increased by germination, enhancing the antioxidant capacity (42).

**TABLE 4:** Correlation analysis of free polyphenols (FP) and flavonoids (FF) content and antioxidant activity of mung bean sprouts.

Indicators	Flavonoid	Polyphenol	*T*-AOC	DPPH RSC	ABTS RSC
Flavonoid	1				
Polyphenol	0.998**	1			
*T*-AOC	0.993*	0.984*	1		
DPPH RSC	0.988*	0.978	0.999**	1	
ABTS RSC	0.962	0.946	0.988*	0.993*	1

*Significant (*p* < 0.05), **extremely significant (*p* < 0.01). *T*-AOC, total antioxidant capacity; ABTS, 2,2-Azino-bis-3-etilbenzotiazolin-6-sulfonic acid; DPPH, 2,2-diphenyl-1-picrylhydrazyl; RSC, radical scavenging capacity.

### 3.5. Differences in metabolites of mung bean sprouts

**FIGURE 5:**
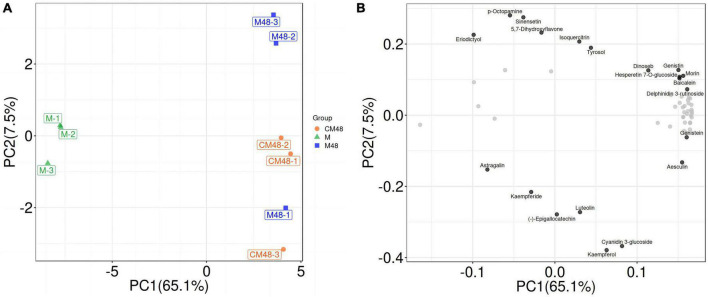
Untargeted metabolomics PCA score plot **(A)** and PCA-Loading plot **(B)**. M, ungerminated mung bean; CM48, mung bean germinated for 48 h; M48, mung bean pretreated with a combination of ultrasonic and γ-aminobutyric acid (GABA) and germinated for 48 h.

On the PCA score graph, the QC samples were clustered together, indicating that the results are highly reliable and reproducible, and the systematic error is within the controllable range (Figure 5). Different germination treatments were separated, and there was a clustering trend within the group. The analysis showed differences in metabolites of mung beans for different germination treatments. The untargeted metabolomics results also showed differences in the content and composition of polyphenols during the germination of mung beans treated by a combination of ultrasound with GABA. These differences are resulted from accumulation and metabolism of polyphenols metabolites during processing.

**FIGURE 6:**
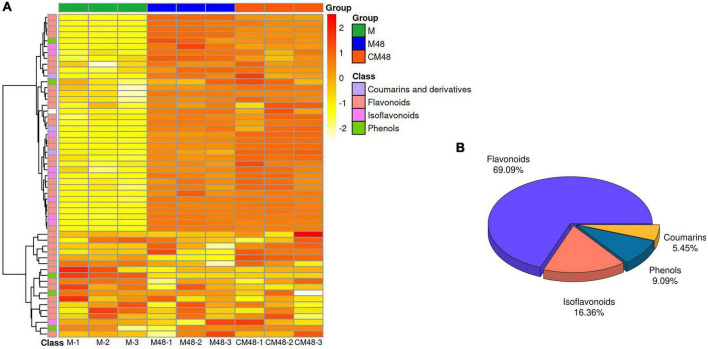
Polyphenol metabolites heat map **(A)** and component ratio chart **(B)** of mung bean sprouts. M, untreated mung bean; CM48, mung bean germinated for 48 h; M48, mung bean pretreated with ultrasonic and γ-aminobutyric acid (GABA) and germinated for 48 h.

The ultrasonic pretreatment resulted in differences in the composition of metabolites in mung bean sprouts of different germination stages with exogenous GABA as a nutrient solution (Figure 6). About 55 polyphenolic metabolites were identified using non-targeted metabolomics techniques (Figure 6A). The identified polyphenol compounds can be divided into four categories, flavonoids, isoflavonoids, phenols, coumarins, and derivatives (Figure 6B).

**FIGURE 7:**
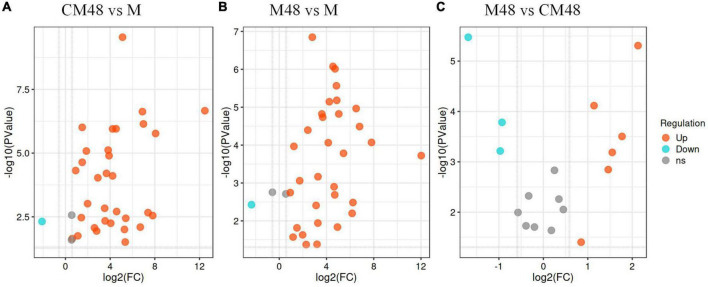
Volcano plot depicting up- and down- accumulated metabolites for each pairwise comparison. M, ungerminated mung bean; CM48 vs. M **(A)**, M48 vs. M **(B)**, and M48 vs. CM48 **(C)**; CM48, mung bean germinated for 48 h; M48, mung bean pretreated with a combination of ultrasonic and γ-aminobutyric acid (GABA) and germinated for 48 h.

Differential accumulation of polyphenolic compounds in mung beans treated with a combination of ultrasonic and GABA and germinated for 48 h (M48), mung bean germinated for 48 h without ultrasonic and GABA treatment (CM48), and untreated mung bean seeds (M), and the results are shown on a volcano plot (Figure 7). CM48 had 31 upregulated and 1 downregulated polyphenolic compound compared to M (Figure 7A). However, M48 showed 32 upregulated and 1 downregulated polyphenolic compound compared to M (Figure 7B). Additionally, M48 showed 6 upregulated [Genistein, (-)-Epigallocatechin, Epicatechin, Nobiletin, Naringenin, Biochanin A] and 3 downregulated [Delphinidin 3-rutinoside, Morin, (2S)-Liquiritigenin] polyphenolic compounds compared to CM48 (Figure 7C).

**FIGURE 8:**
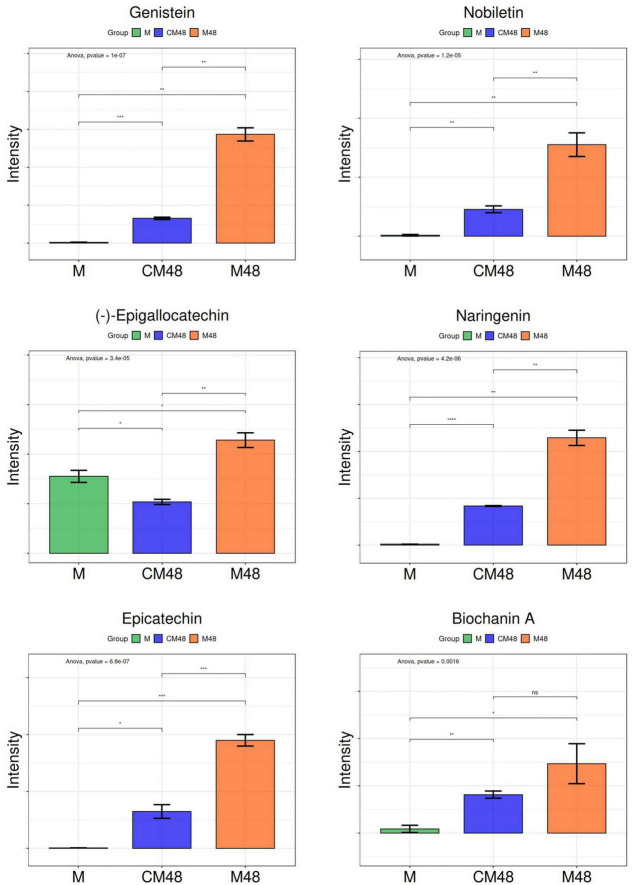
Changes in polyphenols of germinated mung beans. M, untreated mung bean; CM48, mung bean germinated for 48 h; M48, mung bean pretreated with a combination of ultrasonic and γ-aminobutyric acid (GABA) and germinated for 48 h.

Figure 8 shows the relative content of six metabolites found in mung bean treated by a combination of ultrasound with GABA and germinated for 48 h. Genistein has been found to possess beneficial effects by inhibiting the migration of tongue cancer cells, preventing women’s diseases and regulating intestinal health (43–45). (-)-Epigallocatechin and Epicatechin can be used as adjuvants in the treatment of hemostatic disorders caused by snake venom, and also have retinal and cardiovascular health effects (46–48). Nobiletin improves diabetic nephropathy, regulates platelet function, and reduces non-alcoholic fatty liver disease (49–51). Naringenin is an effective anti-cancer agent that reduces inflammation and allergic reactions (52, 53). Biochanin A can effectively lower blood lipids (54).

### 3.6. Metabolic pathway analysis

**FIGURE 9:**
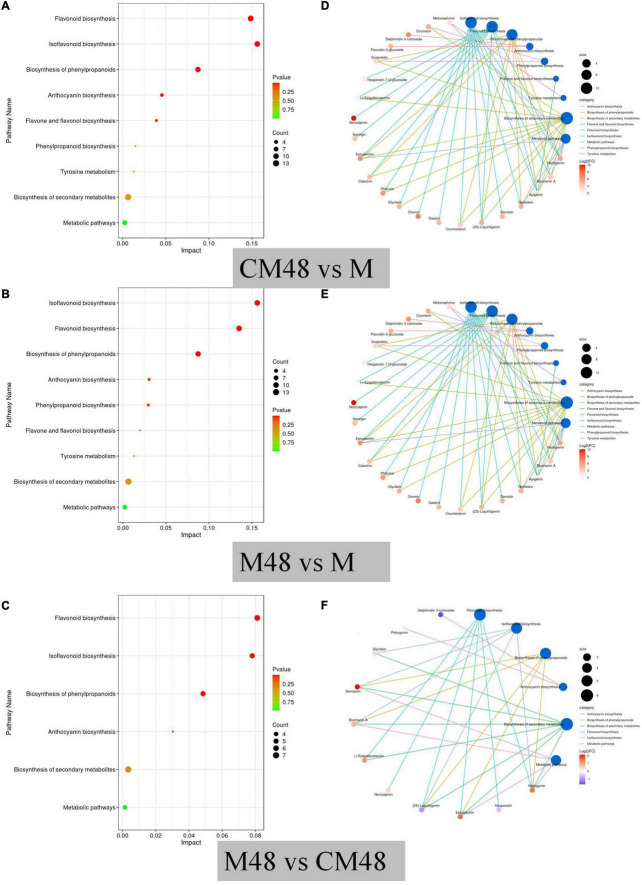
KEGG pathway enrichment of differential metabolites **(A–C)**, and network diagram of the distribution of differential metabolites in metabolic pathways **(D–F)**. M, ungerminated mung bean; CM48, mung bean germinated for 48 h; M48, mung bean pretreated with a combination of ultrasonic and γ-aminobutyric acid (GABA) and germinated for 48 h.

To explore the main metabolic pathways, differential polyphenol metabolites were annotated based on the KEGG database. The results showed that the main metabolic pathways of polyphenol metabolites in mung beans at different germination modes were isoflavones biosynthesis, flavonoid biosynthesis, biosynthesis of phenylpropanoids, anthocyanin biosynthesis, phenylpropanoid biosynthesis, flavone and flavonol biosynthesis, tyrosine metabolism, biosynthesis of secondary metabolites, and metabolic pathways (Figures 9A, B). The most important metabolic pathways were flavonoid biosynthesis, isoflavones biosynthesis, and biosynthesis of phenylpropanoids. Analysis of the differences in metabolic pathways between mung bean treated by a combination of ultrasound with GABA and germinated for 48 h and mung bean germinated without ultrasonic and GABA treatments (Figure 9C) revealed that six metabolic pathways were activated, including flavonoid biosynthesis, isoflavones biosynthesis, biosynthesis of phenylpropanoids, anthocyanin biosynthesis, biosynthesis of secondary metabolites, and metabolic pathways. Figures 9D–F shows distribution of the differential metabolites along the metabolic pathway between the different contrasts. From Figures 9D, E, it can be seen that both control germination and germination combined with ultrasound and GABA treatments were effective in activating polyphenol metabolic pathways. Figure 9F shows the differences in metabolic pathways between germination combined with ultrasound and GABA treatments and control germination groups, six metabolic pathways were enhanced after pretreatment. These findings suggest that pretreatment with a combination of ultrasound and exogenous GABA enhance the accumulation of polyphenols during mung bean germination.

## 4. Conclusion

Mung bean sprouts enriched with polyphenols were produced using a combination of ultrasound and exogenous GABA pretreatment. Ultrasound power of 370 W, ultrasound time of 40 min, and GABA concentration of 10 mmol/L were found to be optimal for pretreatment of mung bean seeds. The combined ultrasound and GABA pretreatment enhanced the content of free polyphenols and flavonoids and antioxidant capacity of the germinated mung beans compared to germination without pretreatment. Results of the untargeted metabolomic analysis indicated that a combination of ultrasound and exogenous GABA pretreatment significantly enhanced the content of six polyphenols in the germinated mung beans. The ultrasonic treatment at optimal power and time softened coat of mung bean seeds and enhanced the absorption of GABA solution. Besides, the cavitation effect of ultrasound produced tiny pores in the seed cell wall, which facilitated membrane transport of GABA. The GABA was involved in the flavonoids, isoflavones, and phenylpropanoids synthesis, and metabolic pathways. The appropriate intensity of ultrasound treatment enhanced the permeability of the cell wall and accelerated the transmembrane transport of GABA. GABA acted as an enzyme activator and promoted the metabolism of polyphenols. Further research is needed to investigate potential health benefits of the mung bean sprouts in animal models and human subjects.

## Data availability statement

The original contributions presented in this study are publicly available. This data can be found here: https://figshare.com/articles/dataset/Untargeted_metabolic_data_xlsx/21431454/1.

## Author contributions

LW and XL: conceptualization. SL and FG: methodology. XL: software and formal analysis. LW, XL, and FG: validation. XL, SL, and YL: investigation. CW and LW: resources and funding acquisition. SL and YL: data curation and visualization. XL and FG: writing—original draft preparation. LW and FG: writing—review and editing. LW: supervision and project administration. All authors read and agreed to the published version of the manuscript.

## Footnotes

1
http://www.hmdb.ca

2
http://www.massbank.jp/

3
http://www.lipidmaps.org

4
https://www.mzcloud.org

5
http://www.genome.jp/kegg/
